# Inflammation-related Proteins Support Diagnosis of Inflammatory Bowel Disease and Are Modified by Exclusive Enteral Nutrition in Children With Crohn’s Disease, Especially of Ileal Phenotype

**DOI:** 10.1093/ibd/izae107

**Published:** 2024-06-26

**Authors:** Bernadette White, Vaios Svolos, Lisa Gervais, Aleksandra Jatkowska, Ben Nichols, Jonathan MacDonald, John Paul Seenan, Richard Hansen, Richard K Russell, Simon Milling, Konstantinos Gerasimidis

**Affiliations:** Department of Human Nutrition, College of Medicine, Veterinary and Life Sciences, University of Glasgow, Glasgow Royal Infirmary, Glasgow, United Kingdom; Department of Human Nutrition, College of Medicine, Veterinary and Life Sciences, University of Glasgow, Glasgow Royal Infirmary, Glasgow, United Kingdom; Department of Paediatric Gastroenterology, Royal Hospital for Children, Glasgow, United Kingdom; Department of Human Nutrition, College of Medicine, Veterinary and Life Sciences, University of Glasgow, Glasgow Royal Infirmary, Glasgow, United Kingdom; Department of Human Nutrition, College of Medicine, Veterinary and Life Sciences, University of Glasgow, Glasgow Royal Infirmary, Glasgow, United Kingdom; Department of Gastroenterology, Queen Elizabeth University Hospital, Glasgow, United Kingdom; Department of Gastroenterology, Queen Elizabeth University Hospital, Glasgow, United Kingdom; Division of Clinical and Molecular Medicine, School of Medicine, University of Dundee, Dundee, United Kingdom; Department of Paediatric Gastroenterology, Royal Hospital for Children & Young People, Edinburgh, United Kingdom; School of Infection & Immunity, College of Medical, Veterinary, and Life Sciences, University of Glasgow, Glasgow, United Kingdom; Department of Human Nutrition, College of Medicine, Veterinary and Life Sciences, University of Glasgow, Glasgow Royal Infirmary, Glasgow, United Kingdom

**Keywords:** Crohn’s, ulcerative colitis, nutrition, Olink

## Abstract

**Background:**

The immunological effects of treatment with exclusive enteral nutrition (EEN) in Crohn’s disease (CD) remain unknown. We characterized the plasma levels of inflammation-related proteins (IRPs) in children with CD and ulcerative colitis (UC) compared with noninflammatory controls (non-IBD) and explored the effect of EEN in CD.

**Methods:**

Ninety-two IRPs were quantified using Olink proteomics in children with CD (*n* = 53), UC (*n* = 11), and non-IBD (*n* = 19). For 18 children with active CD, IRPs were measured before and after 8 weeks of EEN. Relationships with disease phenotype and response to EEN were studied.

**Results:**

Compared with non-IBD, patients with active UC and CD had different levels of 27 (24 raised, 3 decreased) and 29 (26 raised, 3 decreased) IRPs, respectively. Exclusive enteral nutrition modified the levels of 19 IRPs (13 increased, 6 decreased including CCL23, interleukin-24, interleukin-6, and MMP-1). More pronounced changes in IRP profile were observed in patients with ileal involvement and a ≥50% decrease in fecal calprotectin during EEN compared with those with colonic involvement and a <50% decrease in fecal calprotectin, respectively. A machine-learning model utilizing baseline IRP profile predicted response to EEN with a sensitivity of 89%, specificity of 57%, and accuracy of 73%. Thymic stromal lymphopoietin was the most important IRP in the model, this being higher in responders.

**Conclusions:**

Inflammation-related proteins may be useful in the differential diagnosis of IBD. Exclusive enteral nutrition extensively modulated IRPs levels in children with active CD with more pronounced effects observed in patients who showed a reduction in FC and had ileal disease involvement.

## Graphical Abstract

**Graphical Abstract:**
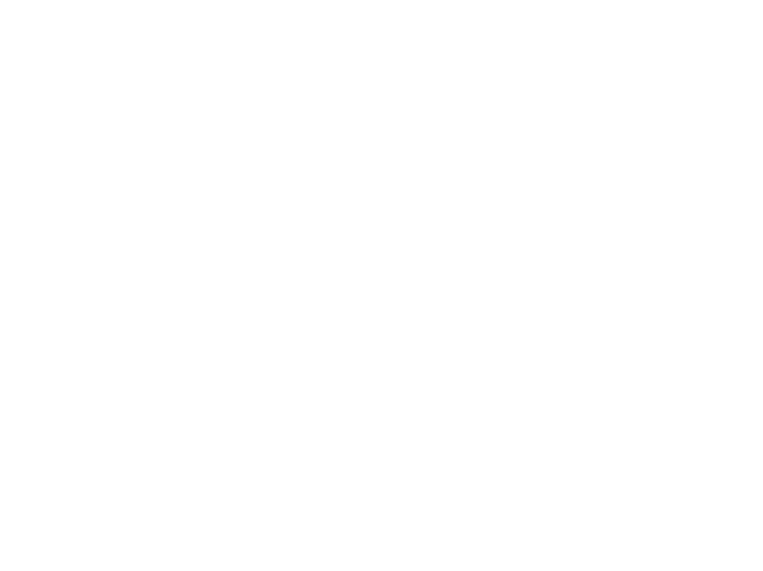

**Key Messages**

**What is already known?** Exclusive enteral nutrition (EEN) is an effective treatment of active Crohn’s disease (CD), but there is little research into how it modulates immune responses.

**What is new here?** Inflammation-related protein profile differs between children with CD and ulcerative colitis and compared with noninflammatory controls. Exclusive enteral nutrition extensively modulates inflammation-related protein levels in active CD with more pronounced effects observed in patients who show a reduction in fecal calprotectin and have ileal disease involvement.

**How can this study help patient care?** Inflammation-related protein profiles could aid in the differential diagnosis of IBD and help to stratify patients for treatment with EEN.

## Introduction

Ulcerative colitis (UC) and Crohn’s disease (CD) are chronic inflammatory conditions of the gastrointestinal tract.^1^ Exclusive enteral nutrition (EEN) for 6 to 8 weeks is recommended as first-line treatment for active CD in children.^2^ Several studies, including meta-analyses, have shown EEN to be at least as effective as oral corticosteroids in inducing clinical remission but with a superior safety profile, whilst also improving markers of nutritional status and promoting mucosal and transmural healing.^3,4^

Despite the increasing breadth of evidence for the efficacy of EEN, there is a scarcity of research exploring how treatment with EEN works and how it modifies immune response. Further to this, whether blood proteomic or metabolomic signatures prior to and during EEN can predict patients whose disease markers and activity will improve has also not been studied extensively in the literature. Yamamoto et al^5^ measured cytokine concentrations in mucosal biopsies from patients with active CD before and after 4 weeks of EEN with elemental formula and compared these to samples from healthy controls. Prior to treatment initiation, the levels of Interleukin (IL) 8, IL-6, and transforming growth factor alpha (TGF- α), were higher than controls, whereas after 4 weeks of treatment, 71% of participants achieved remission and cytokine levels decreased to levels similar to the control population.^5^ In contrast, Rolandsdotter et al found no significant changes in the mucosal cytokine profiles of 5 children with CD and 1 child with inflammatory bowel disease (IBD)-unclassified after treatment with EEN.^6^ We previously investigated changes in cytokine concentration during 8 weeks of EEN in peripheral blood samples of children with active CD. Patients who entered clinical remission had a significant decrease in IL-6, IL-17E, IL-17F, and IL-31.^7^ This observation paralleled findings observed by Schwerd et al in which peripheral blood mononuclear cells extracted from pediatric patients with CD after 3 weeks of EEN, when stimulated with bacterial antigens, had lower expression of inflammatory cytokines, particularly IL-6, IL-8, IL-1 β, and T helper (Th) 1–derived interferon-gamma (IFN-γ).^8^

Proximity extensions assay (PEA) is a form of high-throughput immunoassay that works through the binding of antibodies to the surface of the target protein which has a specific single-stranded nucleotide tag that hybridizes when another antibody with a complementary sequence binds the oligonucleotide tag.^9^ These oligonucleotide sequences are then subjected to real-time quantitative polymerase chain reaction (PCR) to allow for the quantification of the bound target protein.^9^ Recently, Bourgonje et al^10^ profiled the levels of 92 inflammation-related proteins (IRPs) using PEA in 1028 IBD patients and 148 healthy controls. Following adjustments for age, sex, and body mass ineex (BMI), 24 and 20 proteins were different in active CD and UC, respectively, compared with healthy controls. For patients with IBD in clinical remission, 15 proteins in CD and 12 in UC remained significantly different from healthy controls. In both patients with CD and UC, the inflammatory cytokines oncostatin M (OSM) and IL-6 were increased, as well as the chemotactic protein IL-8. In reverse, the proteins delta and notch-like epidermal growth factor-related receptor (DNER), which is involved in the Notch-signaling pathway, and fibroblast growth factor FGF-19 were decreased in patients with CD and UC compared with healthy controls. Certain protein biomarkers also varied according to disease phenotype with lower levels of FGF-19 observed in patients with CD with ileal disease or prior bowel surgery compared with those with colonic disease.^10^ Similar results were observed by Andersson et al^11^; FGF-19 was lower in CD with ileal/ileocolonic phenotype compared with UC. Based on the protein profiles quantified as part of this study, patients with CD and UC could be distinguished from healthy controls with 88% and 81% accuracy, respectively.^11^

Kalla et al identified 15 proteins, including IL-6 and OSM which function independently of tumor necrosis factor (TNF) signaling, which were significantly associated with the need for treatment escalation in patients with IBD.^12^ Recently, a study^13^ explored the effect of conventional treatment with EEN or oral prednisolone against infliximab on 92 IRPs in children with CD. Far fewer proteins were modulated by EEN than infliximab; nonetheless, compliance to treatment with EEN in this previous study was not assessed with an objective biomarker,^14^ with samples collected up to 6 weeks’ post-EEN completion, when the majority of patients experience a rapid increase in biomarkers of gut inflammation, suggestive of early signs of disease relapse.^7^ Changes according to response to treatment were not reported either.

In the present study, our primary objective was to explore the effect of EEN on IRP profile and associate changes observed with disease location and response to treatment. Prior to doing this, we also investigated the usefulness of differential IRPs in aiding the diagnosis of IBD.

## Materials and Methods

### Eligible Participants

**Figure 1.**
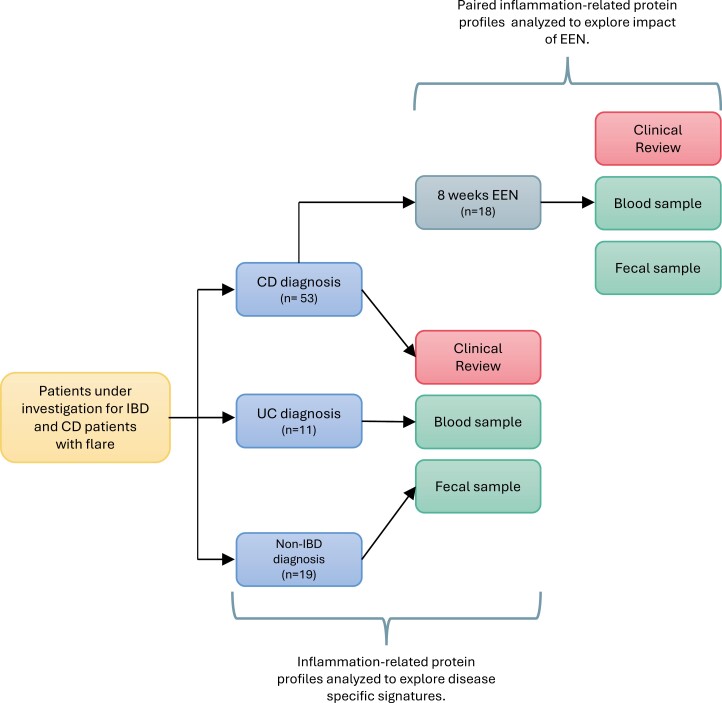
Schematic flow chart of study design and participation. Abbreviations: IBD, Inflammatory bowel disease; CD, Crohn’s disease; UC, Ulcerative colitis; non-IBD, non-inflammatory bowel disease; EEN, exclusive enteral nutrition.

Children who visited the Royal Hospital for Children in Glasgow and underwent investigations for IBD diagnosis were recruited prospectively. Patients were diagnosed with IBD based on standard clinical, endoscopic, and histological findings.^15^ Disease behavior and location were classified using the Paris classification.^16^ Patients with absence of intestinal pathology following endoscopy with biopsy were classified as a group of noninflammatory controls (non-IBD). The primary study group was patients with CD, whereas patients with UC or non-IBD were used for comparative analysis of IRPs at diagnosis (Figure 1).

Patients diagnosed with treatment-naïve active CD were prescribed EEN (Modulen, IBD, Nestle, Vevey Switzerland) for 8 weeks as their sole standard of care treatment, and as described previously,^7^ treatment with EEN covered patients’ dietary recommendations with undernourished children (BMI Z-score < −2) prescribed a 10% higher energy intake.^17^ Throughout treatment with EEN, patients were not allowed to consume any other food except for water, coffee, clear mints, and syrup flavoring. Patients were encouraged to consume EEN orally but if patients could not tolerate their prescribed volumes within the first 3 days, they were switched to nasogastric tube feeding. In addition to patients with treatment-naïve CD, patients who had previously received a CD diagnosis and were to receive EEN for disease relapse were recruited too. Patients were allowed to continue their background therapy with immunomodulator as directed by the caring medical team. Children under the age of 3 years old and those who received antibiotics in the preceding month were excluded.

### Disease Activity

For children with CD undertaking treatment with EEN, clinical disease activity was assessed using the weighted pediatric Crohn’s disease activity index (wPCDAI).^18^ Assessments were performed prior to treatment initiation and at the end of EEN by the caring clinician. Clinical remission was defined as a wPCDAI score of ≤12.5. C-reactive protein (CRP) was measured at treatment initiation and at the end of EEN.

### Fecal Calprotectin and Gluten Immunogenic Peptide

Fecal samples were collected from patients at diagnosis or EEN initiation and completion. The whole bowel movement was collected and homogenized within 4 hours of passage and stored at −80°C until analysis. The concentration of fecal calprotectin (FC) and of fecal gluten immunogenic peptide (GIP), a proxy of compliance to EEN,^14^ were determined using the CALPROLAB0170 (ALP) ELISA kit (Lysaker, Norway) and the iVYDAL In Vitro Diagnosis iVYLISA GIP-S kit (Biomedal S L., Seville, Spain), respectively, following manufacturer instructions. The lower limit of detection of the FC assay is 25 mg/kg. Patients in whom FC decreased >50% during EEN were classed as FC responders; those with a <50% decrease in FC were classed as FC nonresponders. A 50% decrease in FC was selected as a cut-off, as this has previously been shown to be a predictive factor for inactive endoscopic disease in pediatric patients with CD on EEN.^19^ Fecal GIP concentrations were classed in a binary manner with those patients with an undetectable concentration classified as “negative” and EEN compliant and those with a detectable concentration classified as “positive” and non-EEN compliant.

### Inflammation-related Protein Analysis

Blood samples were collected in lithium heparin tubes from patients at treatment initiation (prior to endoscopy) and at treatment completion, after 8 weeks of EEN. Blood samples were centrifuged at 400 *g* for 20 minutes at room temperature to separate plasma from cells. Plasma samples were shipped to Olink, Uppsala, Sweden, where analysis of 92 IRPs was carried out using the Olink Inflammation Panel which utilizes PEA technology.

### Statistical Analysis

Statistical analysis was performed using Minitab version 19 (State College, PA, USA), GraphPad Prism V9 (Boston, MA, USA), and R version 4.2.1 (R Foundation for Statistical Computing, Vienna, Austria). Relationships between the 92 IRPs with age, height, weight, and BMI Z-scores were explored in the non-IBD group to unveil physiological changes. General linear models were performed to first assess differences between groups with IBD compared with non-IBD controls. Independent of this analysis, general linear model analysis was performed in patients with CD who had paired samples available to explore the effect of treatment with EEN and to assess the interaction of thiopurines with IRPs. Post hoc Bonferroni correction was applied to correct for multiple testing. Machine-learning with random forest analysis and recursive feature elimination was carried out on R^20^ to explore if levels of IRPs and FC can distinguish different forms of IBD from each other and from non-IBD controls and to explore if baseline levels and changes in IRP levels could predict FC response to EEN.

### Ethical Permissions and Power Calculation

Approval for this study was obtained from NHS West of Scotland Research Ethics Committee (14/WS/1004), and the study was registered in clinicaltrials.gov (NCT02341248). The main study was powered (*n* = 42 patients with CD) to detect differences in microbial metabolites during the induction phase of active CD with EEN.^7^ In this substudy, we used all available baseline samples for patients with UC, CD, and non-IBD and then all available paired baseline and end of treatments samples for patients with CD who completed EEN.

## Results

### Participant Characteristics

In total, 83 patients were recruited (53 CD,11 UC, and 19 non-IBD; Table 1). Patients with UC were older, and patients with CD had a lower weight and BMI Z-score than non-IBD controls. Eighteen patients with CD received EEN therapy and provided paired blood samples at treatment initiation and follow-up; of these, 10 patients with CD were on no other treatment, and 8 started thiopurines during their 8-week treatment with EEN.

**Table 1.** Baseline characteristics of patients at study enrollment.

	Crohn’s Disease		Ulcerative Colitis		Non-IBD
**Characteristic**					
Number of patients, *n*	53		11		19
Number previously diagnosed, *n*	3		N/A		N/A
Age, median (Q1, Q3), years	13.7 (10.6, 14.8)		14.6 (13.2, 15.0)^*^		11.8 (10.7, 13.2)^*^
Females, *n* (%)	18 (34.0)		5 (45.5)^*^		3 (15.8)^*^
Height Z-score (SD)	−0.19 (1.12)		−0.38 (1.17)		−0.09 (0.91)
Weight Z-score (SD)	−0.58 (1.04)^†^		−0.50 (2.03)		0.33 (1.11)^†^
BMI Z-score (SD)	−0.90 (1.28)^†^		−0.64 (2.30)		0.55 (1.17)^†^
Faecal Calprotectin (μ g/g), median (Q1, Q3)	1452 (727)^†^		1672 (740)^*^		45.9 (126)^†*^
**Paris Disease Classification**					
Age at diagnosis (%)					
	**A1a (0 to <10)**	**13 (24.1)**			
	**A1b (10 to < 17)**	**40 (74.1)**			
**Disease location (%)**					
	L1	3 (5.6)	E1	0	
	L1, L4a	1 (1.9)	E2	2 (18.2)	
	L2	9 (16.7)	E3	0	
	L2, L4a	7 (13.0)	E4	9 (81.8)	
	L2, L4b	1 (1.9)			
	L2, L4a, L4b	1 (1.9)			
	L3	8 (14.8)			
	L3, L4a	9 (16.7)			
	L3, L4b	2 (3.7)			
	L3, L4a, L4b	12 (22.2)			
	Perianal	4 (7.4)			
**Disease behavior (%)**					
	B1	53 (98.1)			
**Medications in patients with Crohn’s disease on EEN (n = 18)**	**Baseline**		**After 8 weeks**		
No medication, n (%)	10 (55.5)		6 (33.3)		
Azathioprine, n (%)	0		7 (38.9)		
6-Mercaptopurine, n (%)	0		1 (5.5)		
Mesalamine, n (%)	1 (5.5)		0		

Data are displayed with mean (SD) unless otherwise stated; ^*^P ≤ .05 between patients with ulcerative colitis and non-IBD controls, ^†^*P* ≤ .05 between patients with Crohn’s disease and non-IBD controls.

Abbreviations: non-IBD, noninflammatory bowel disease; SD, standard deviation.

### Inflammation-related Proteins Relate to Age and Anthropometric Characteristics in Non-IBD Controls

Correlations between the 92 IRPs with age, height, weight, and BMI Z-scores were investigated in the non-IBD control group. Of these, 10 proteins correlated with age, 8 proteins correlated with BMI Z-score, 10 proteins correlated with weight Z-score, and 8 proteins correlated with height Z-score (Supplementary Figure 1). For these IRPs, age and anthropometry were adjusted as covariates in subsequent differential analyses between patient groups.

### Inflammation-related Proteins Are Predictive of IBD Diagnosis and Disease Type

**Figure 2.**
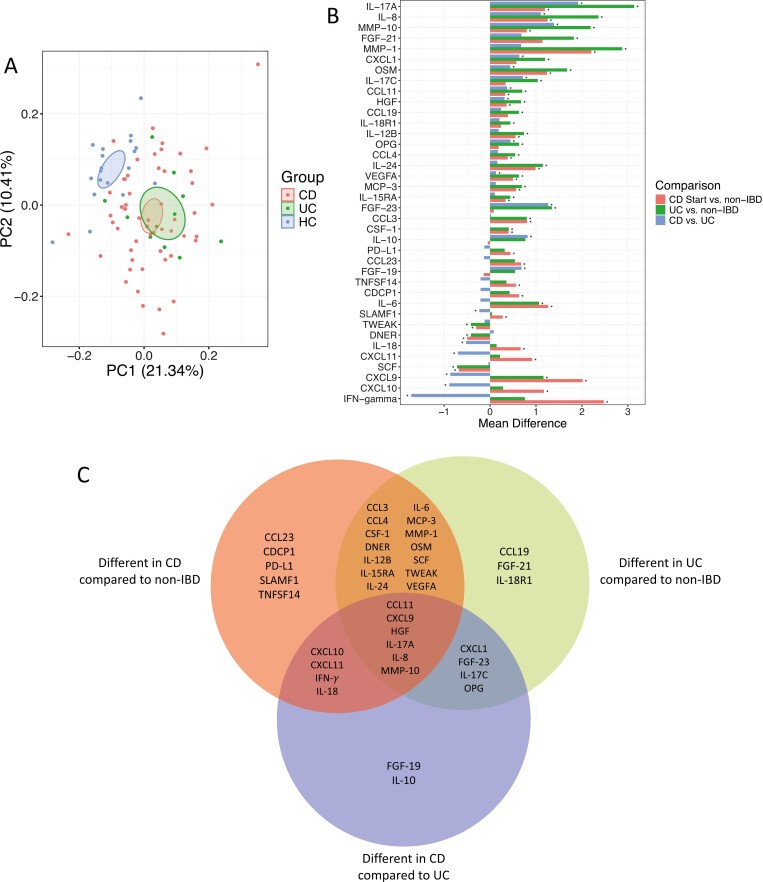
A, Principal component analysis of inflammation-related protein profiles of patients with Crohn’s disease, ulcerative colitis, and non-IBD controls. B, Bar chart of inflammation-related proteins which were significantly (*P* ≤ .05) different in patients with inflammatory bowel disease (IBD) compared with non-IBD controls, as well as between patients with Crohn’s disease (CD) and ulcerative colitis (UC). In all cases, the second group presented is the control the other was compared to. *P ≤ 0.05. C, Venn diagram of the proteins found to be significantly different between each IBD subtype compared to non-IBD controls and between each IBD subtype.

Prior to investigating the effect of EEN in children with CD, differences in IRP profiles were explored between children with IBD and non-IBD controls. In principal component analysis (PCA), IRP profiling separated patients with CD (R^2^ = 0.11%, *P* = .001) and UC (R^2^ = 0.22%, *P* = .001) from non-IBD controls. Inflammation-related protein profile clustered differently in patients with CD from patients with UC, although the proportion of variance explained (R^2^ = 0.046%, *P* = .001) was less than when either of the groups were compared against non-IBD controls (Figure 2A).

Compared with non-IBD controls, patients with UC and CD had higher levels for 24 and 26 proteins, respectively; 17 of which overlapped between the 2 groups (Figure 2B, C, Supplementary Figure 2). Proteins at higher levels in patients with IBD included those within the neutrophil and Th17 cell pathways such as OSM, IL-6, IL-8, and IL-17A. In contrast, DNER, stem cell factor (SCF), and tumor necrosis factor-like weak inducer of apoptosis (TWEAK) were at lower levels in patients with IBD compared with non-IBD controls. These 3 proteins have roles in cell survival and proliferation^21-23^ (Figure 2B, C, Supplementary Figure 2).

Sixteen IRPs differed between patients with CD and those with UC (Figure 2B, C, Supplementary Figure 2). Proteins observed to be at lower levels in patients with UC compared with CD were those associated with IFN-γ and Th1 cell pathways, with 4 of these 5 (80%) proteins (IFN-γ, CXCL10, CXCL11, IL-18) also higher in patients with CD compared with non-IBD controls—but not statistically different between patients with UC and non-IBD controls (Supplementary Figure 2). Conversely, 11 proteins observed to be at higher levels in patients with UC compared with CD were related to a variety of different pathways with an overarching theme of regulatory roles such as IL-10, a potent anti-inflammatory cytokine, and FGF-19 and FGF-23, which each regulate different aspects of metabolism and gut physiology.^24^

There were 32 (15 positive, 17 negative) correlations with CRP and 22 (20 positive, two negative) correlations with FC and IRPs patients with CD at baseline (Supplementary Figure 3). In patients with UC, 2 proteins correlated positively with CRP and 1 with FC (Supplementary Figure 3).

**Figure 3.**
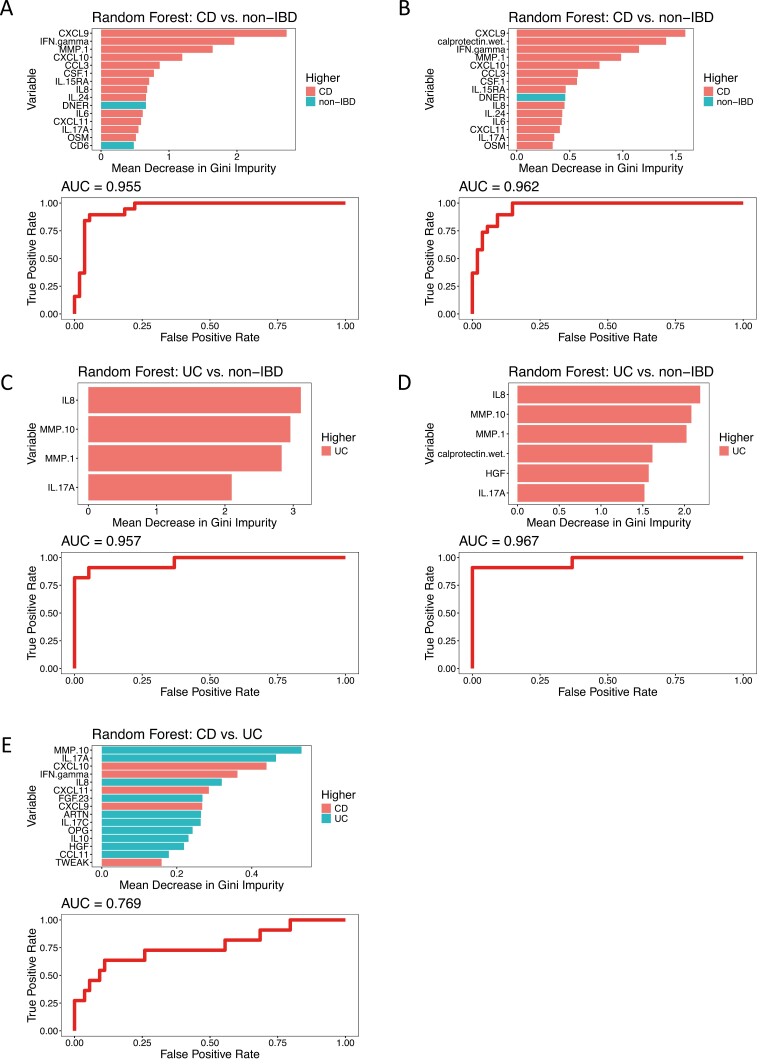
Bar chart and receiver operating characteristic analysis from the random forest modelling with recursive feature elimination to distinguish different patient groups. A, Model to distinguish patients with Crohn’s disease (CD) from noninflammatory bowel disease(non-IBD) controls using only inflammation-related proteins (IRPs). B, Model to distinguish patients with CD from non-IBD controls using IRPs and fecal calprotectin (FC). C, Model to distinguish patients with ulcerative colitis (UC) from non-IBD controls using only IRPs. D, Model to distinguish patients with UC from non-IBD controls using IRPs and FC. E, Model to distinguish patients with CD from patients with UC using only IRPs. Abbreviation: AUC, area under the curve

A series of random forest models with recursive feature elimination were performed to identify a minimal set of features able to distinguish between CD and non-IBD (Figure 3A, B), UC and non-IBD (Figure 3C, D), and CD and UC (Figure 3E). Models to distinguish between patients with CD and non-IBD controls had an out-of-bag error rate (OOB) of 10.7% (accuracy of 89%) and presented the same sensitivity (90%) and specificity (89%) whether FC levels were included (Figure 3B) or not (Figure 3A) in the model. In these models, CXCL-9, IFN- γ, and MMP-1 were the most influential IRPs to differentiate CD patients from non-IBD controls (Figure 3A), all of which were higher in patients with CD than non-IBD controls. When FC was added to the model (Figure 3B), CXCL-9 and IFN- γ remained the most influential IRPs alongside FC, all of which were higher in patients with CD than non-IBD controls.

Models to distinguish between patients with UC and non-IBD controls performed slightly better when FC measurements were included with an OOB of 6.7% (accuracy of 93%) with FC inclusion and 10% (accuracy of 90%) without FC inclusion (Figure 3C, D). The addition of FC in the model increased the sensitivity to 91% from 82%, although the specificity remained unchanged (95%). Interleukin-8, MMP-10, and MMP-1 had the largest influence in prediction in both models, all of which were at higher levels in patients with UC than non-IBD controls.

When trying to distinguish between patients with CD and UC (Figure 3E), only the model including IRPs was successful with an OOB of 17% (accuracy of 83%) with a sensitivity and specificity of 82% and 87% respectively; MMP-10, IL-17A, CXCL10, and IFN-γ contributed the most to the prediction; the first 2 were at higher levels in patients with UC, and CXCL10 and IFN-γ were at higher levels in patients with CD. These models highlight the role of IFN-γ associated pathways and Th1 cells in the pathogenesis of CD more so than in UC as has been previously highlighted in the literature.^25,26^ Alternatively, we saw more of a role for Th17 cells in the pathogenesis of UC.

### Exclusive Enteral Nutrition Extensively Modulates Inflammation-related Proteins in the Blood of Children with CD

**Figure 4.**
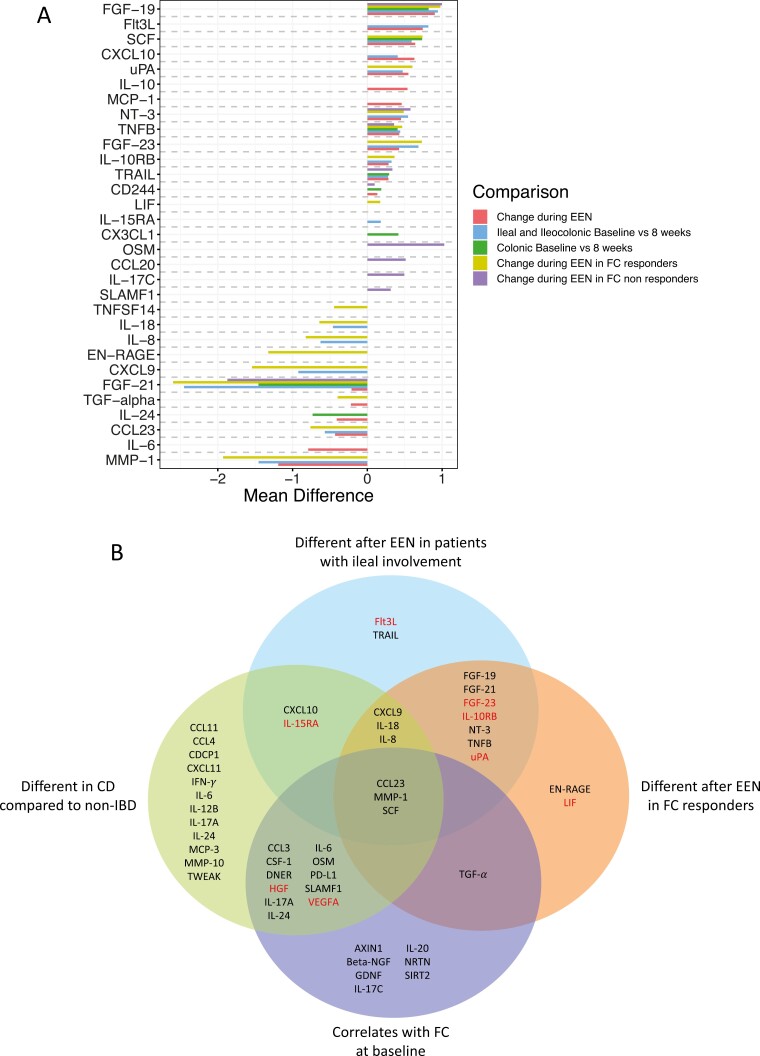
A, Bar chart of the inflammation-related proteins (IRPs) shown to have a significant (*P* ≤ .05) mean difference from baseline after 8 weeks of exclusive enteral nutrition (EEN) in different subsets of patients with Crohn’s disease (CD). Bars are presented only for significant changes found in each comparison group. B, Venn diagram summarizing the main significant (*P* ≤ .05) differences or correlations found in IRPs. Proteins highlighted in red were shown to significantly interact with thiopurines. Abbreviations: non-IBD, noninflammatory bowel disease; FC, fecal calprotectin.

Of the 18 patients with CD on EEN, 13 (72%) entered clinical remission (wPCDAI ≤ 12.5), and of the 16 patients with paired FC measurements available, 9 (56%) demonstrated a baseline decrease of more than 50% at EEN completion and were defined as FC responders. Of those that were classified as FC responders ~78% (7 of 9) tested negative for fecal GIP demonstrating a high level of compliance. Treatment with EEN significantly modified 19 proteins in total, 13 of which significantly increased and 6 significantly decreased (Figure 4A, B, Supplementary Figure 4). Of the 19 IRPs that changed during EEN, 4 (FGF-23, Flt3L, IL-10RB, and uPA) significantly interacted with thiopurine use, meaning their increase was only observed in patients who received concomitant treatment with a thiopurine (Supplementary Figure 5). Four of the 6 proteins (67%) that decreased during EEN (ie, CCL23, IL-24, IL-6, MMP-1) were also higher in patients with CD compared with non-IBD controls prior to EEN initiation. The pathways most likely moderated by these observed decreases were the neutrophil and Th17 cell pathway (CCL23, IL-6 and IL-24) and tissue remodeling (MMP-1).^27-29^ From the 13 proteins that increased during treatment with EEN, 4 different pathways were identified as enhanced, including intestinal physiology (FGF-19, FGF-23), Th1 cells (CXCL10, CD244), control of inflammation (IL-10, IL-10RB), and cell proliferation (TRAIL, SCF). Two IRPs, the CCL23 which decreased and CXCL10 which increased during EEN were different in patients with CD compared with non-IBD controls at treatment initiation. These proteins were not observed to be significantly different between non-IBD controls and the newly diagnosed children with UC.

Of the 18 patients on EEN, 13 had (72%) undetectable GIP after 8 weeks of EEN, meaning that these patients had likely been more adherent to the treatment restrictions and had not yet reintroduced food. In this subset of patients, 16 IRPs were modified by EEN, 12 of which significantly increased and 4 significantly decreased (Supplementary Figure 6). Most of these changes (13 of 16, 81.25%) were seen in the whole cohort of 18 patients.

### Crohn’s Disease Involving the Ileum Is Associated With Extensive Changes in Inflammation-related Proteins in the Plasma of Children With Crohn’s Disease

Changes during 8-week treatment with EEN were explored in patients with CD according to disease location (Figure 4A, B). Patients with ileal and ileocolonic phenotypes were analyzed together as a single group to gain statistical power. Eight IRPs (6 increased, 2 decreased) changed during EEN in patients with isolated colonic disease compared with 17 (11 increased, 6 decreased) proteins that changed in patients with ileal/ileocolonic CD. Five other IRPs (4 increased, 1 decreased) changed during EEN regardless of disease location.

### Improvement of Gut Inflammatory Markers During Exclusive Enteral Nutrition Is Associated With Extensive Changes in Inflammation-related Proteins in the Plasma of Children With Crohn’s Disease

**Figure 5.**
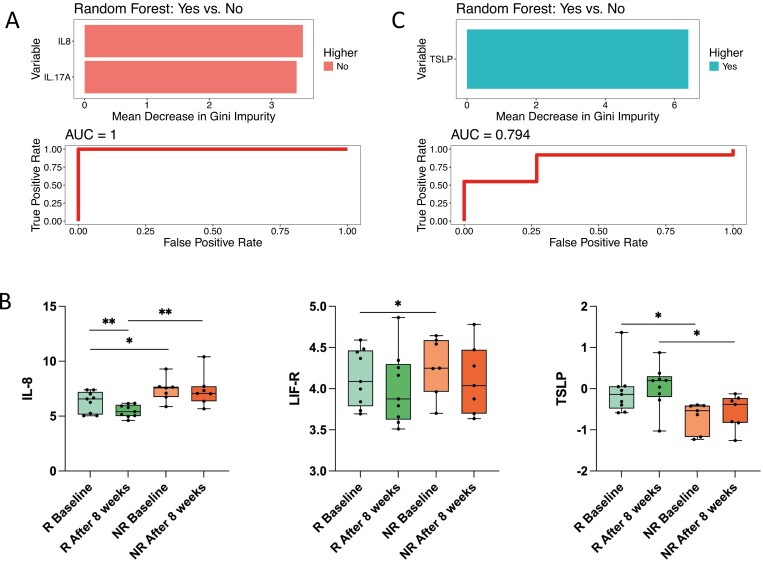
A, Bar chart and receiver operating characteristic analysis from the random forest modelling with recursive feature elimination to determine if the levels of IRPs following 8-week treatment with EEN can predict fecal calprotectin (FC) response to exclusive enteral nutrition (EEN). *Yes* denotes those which do have a ≥50% decrease in FC following 8 weeks of EEN. *No* describes the inverse, those which do not achieve > 0% decrease in FC following 8 weeks of EEN. B, Boxplots of the inflammation-related proteins shown to significantly (*P* ≤ .05) differ in patients with Crohn’s disease, split based on FC response, before 8-week treatment with EEN. *P ≤ .05, **P ≤ .01, ***P ≤ .001. C, Bar chart and receiver operating characteristic analysis from the random forest modelling with recursive feature elimination to determine if baseline IRP levels could predict FC response to EEN. Abbreviations: AUC, area under the curve; R Baseline, Baseline measurement for FC responders; R After 8 week, measurements for FC responders following 8 weeks of EEN; NR Baseline, Baseline measurements for FC nonresponders; NR After 8 weeks, measurements for FC nonresponders following 8 weeks EEN.

To determine if changes in IRP levels differed based on response to EEN, patients were split according to their FC changes from baseline. Fecal calprotectin responders (ie, >50% decrease) had significant alterations in more IRPs, particularly in IRPs that decreased (*n* =19, of which 8 increased and 11 decreased) than FC nonresponders (*n* =12, of which 11 increased and 1 decreased) following EEN (Figure 4A, B, Supplementary Figure 7). Eight of the 11 proteins (73%) that decreased in FC responders were seen in higher levels in patients with CD prior to treatment compared with non-IBD controls. In those proteins that decreased in FC responders, Th1 cell (decrease in CXCL9, IL-18) and neutrophil (decrease in CCL23, EN-RAGE, IL-8) pathways were implicated. Five proteins increased during EEN only in FC responders and 6 only in FC nonresponders. Of note, 3 proteins (NT-3, TNFB, FGF-19) increased and FGF-21 decreased during EEN in both FC responders and nonresponders, suggesting these changes may have been attributed to diet changes during EEN rather than changes related to disease activity or gut inflammation. Similar signals were observed when patients’ response to EEN was assessed using wPCDAI, with more IRPs changing in patients who achieved remission compared with those still with active disease at EEN completion (Supplementary Figure 8). Using random forest analysis with recursive feature elimination, a model utilizing the levels of IRPs at the point of EEN completion had a high sensitivity (100%) and specificity (71%) and OOB of 12% (accuracy of 88%) to classify FC responders from nonresponders (Figure 5A). The levels of IL-8 and IL-17A were higher in those who were FC nonresponders following EEN. Models using the change in IRPs during EEN failed to reach significance.

### Inflammation-related Proteins, Prior to EEN Initiation, Are Predictors of Response to Treatment

Prior to EEN initiation, IRP profiles of FC responders and FC nonresponders were compared to identify differential signals predictive of response to treatment with EEN. Fecal calprotectin responders did not cluster differently to non-FC responders on PCA (*P* = .717); however, FC responders had higher levels of thymic stromal lymphopoietin (TSLP), whereas FC nonresponders had higher levels of both IL-8 and LIF-R. (Figure 5B). A random forest model utilizing baseline levels of IRPs (Figure 5C) achieved a sensitivity of 89%, specificity of 57%, and OOB of 25% (accuracy of 73%) to predict response of EEN, with the baseline levels of TSLP being the most important IRP in predicting response to EEN.

## Discussion

In the present study, several differences were uncovered in IRP profile between patients with IBD compared with our non-IBD cohort, as well as in patients with CD before and after treatment with EEN. In analyses comparing patients with CD and UC to non-IBD controls, distinct profiles were observed in each IBD subtype with IRPs able to differentiate each subtype from non-IBD controls with a consistently high sensitivity and specificity. This highlights the potential of IRP profiling as a tool to aid or complement current biomarkers in the diagnosis of IBD, particularly in CD, thus helping to identify which patients are the right candidates for a colonoscopy.

These profiles also helped to uncover some of the underlining pathological mechanisms, with CD demonstrating enhanced levels of IRPs related to IFN-γ and Th1-associated pathways, whereas in UC we observed an enrichment in IRPs related to Th17 pathways. The role of Th1 cells and IFN-γ pathways in CD has been extensively studied in the literature with an overexpression of IL-18 mRNA^25^ and an upregulation of IFN-γ previously documented in CD, which was not seen in UC.^26^ There has not been a clear population of cells to establish a pathology in UC, but Th2 cells have historically been considered the most likely candidates.^30^ There is increasing evidence for the role of Th17 cells in the pathology of UC, with a murine model of colitis showing that blockade of IL-17 in mice with an upregulated expression of Th17 cells decreased colitis.^31^ Further to this, when mucosal samples from patients with UC and CD were isolated, an increase in IL-17 transcripts in both IBD subtypes was seen compared with controls, more abundant in UC than CD. The same authors showed that recombinant IL-23 enhanced the production of IL-17 in lamina propria CD4+ T cells from patients with UC but had a lesser effect in CD.^32^

In the present study, we were also able to observe changes in the IRP profile following treatment with EEN irrespective of thiopurine use, with a plethora of key pathways modulated including the neutrophil and Th17 cell pathways (CCL23, IL-6, and IL-24), tissue remodeling (MMP-1), intestinal physiology (FGF-19, FGF-23), Th1 (CXCL10, CD244), control of inflammation (IL-10, IL-10RB), and cell proliferation (TRAIL, SCF). The decrease in IL-6 we observed is in agreement with a recent study by Geesala et al. They demonstrated in a rat model of colitis that treatment with EEN eliminated mechanical stress in the colon thereby reducing the mechanotranscription of IL-6, which in turn attenuated the Th17 immune response, thus highlighting how treatment with EEN may induce remission.^33^

In the current study, we also observed a greater modulation of IRP profile after treatment with EEN than in a recent study.^13^ When the authors of the study investigated the impact of first-line infliximab and conventional treatment (EEN for six to 8 weeks or oral prednisolone for four weeks) on 83 IRPs in moderate to severe pediatric patients with CD, 6 proteins (VEGFA, CDCP1, CXCL9, HGF, IL-24, and IFN-γ) were shown to be significantly reduced between baseline and weeks 10 to 14 after treatment with EEN. This is a notably reduced number compared with the 3-fold number of IRP changes (*n* = 19) we observed across our whole population. It has previously been highlighted that as food is reintroduced following treatment with EEN, levels of FC significantly increase drastically after as little as 17 days.^7^ Thus, the reduced number of changes observed by Jongsma et al^13^ may be due to IRP levels being measured after 2 to 8 weeks of food reintroduction, following EEN completion, thus allowing time for these proteins to return to pr-treatment levels. Considering the differential effects treatment with EEN and infliximab have on IRP, and by extension inflammatory pathways, it might be possible that combining both treatments together will demonstrate a higher potency to ameliorate active disease than each treatment in isolation.

When we considered disease location in a subset analysis, more profound changes in IRP profile following EEN were observed in participants, with ileal involvement supporting a potentially better response to EEN in patients with ileal disease involvement.^34^ The impact of EEN in IRP was also observed to be more pronounced in those achieving a ≥50% decrease in FC, with machine-learning being able to use pretreatment IRP profile to predict which participants achieved FC response to EEN. Interleukin-8 and TWEAK at baseline and the levels of IL-8 and IL-17A after treatment with EEN related to those who did not achieve a FC response. There is a breadth of previous studies highlighting raised IL-8 in patients with IBD compared with non-IBD controls in both mucosal biopsies^5,8^ and peripheral blood^10^; therefore, our finding that TSLP is associated with a response to EEN is perhaps a novel finding. Intestinal epithelial cells produce TSLP which has an anti-inflammatory role by inducing the differentiation of Th cells into Th2 cells and modulates the behavior of dendritic cells to both maintain populations of CD4+ T cells and to encourage the differentiation of regulatory T cells.^35^ This highlights the potential of IRPs in patient selection—identifying suitable candidates for treatment with EEN and those likely to be resistant to the therapy who would benefit from an alternative treatment.

Four proteins (NT-3, TNFB, FGF-19, and FGF-21) were found to significantly change following 8-week treatment with EEN regardless of FC response. Two of these, FGF-21 and FGF-19, have clear roles associated with diet, with FGF-19 regulating bile acid homeostasis and FGF-21 regulating energy homeostasis.^24^ Research from the 1000IBD project has previously highlighted FGF-19 to differentially associate with different dietary patterns independent of IBD type and disease activity.^36^ A clear link such as this within patients with IBD between diet and the other 3 IRPs is yet to be proposed; however, indications are present within the literature. For example, the level of FGF-21 has previously been shown to increase following fasting^37^ or a ketogenic diet.^38^ Therefore, it could be hypothesized that the decrease following EEN was because our patients recovered from an undernutrition status. Conversely, TNFB was shown to increase regardless of response to EEN. This protein and other members of its family (particularly TNF- α) have been implicated in diet-induced obesity from knock-out studies in mouse models.^39^ Following 8 weeks of treatment with EEN there was a significant increase in the weight Z-score of our participants; therefore the increase in TNFB could be explained by positive energy balance and weight gain. The most puzzling protein to increase during EEN was NT-3, a protein with roles in both the development of the enteric and peripheral nervous system.^40^ When rats were subjected to a diet of whole or refined grains, the levels of NT-3 decreased in the hippocampus and prefrontal cortex.^40^ As EEN feeds do not contain fiber or gluten, which would be present within grains, we postulate that this could explain an increase in NT-3 within the peripheral blood of our participants, although we have no evidence to support this.

A limitation of the present study is the lack of an independent validation cohort to replicate the signals we observed. Nonetheless, our findings on the importance of IRPs in aiding the differential diagnosis are in general in accordance to previous research carried out mainly in adults with IBD (Supplementary Table 1). Our modest samples size means that we may have been underpowered to demonstrate statistical significance in certain differences between groups. For example, we only had a power of 27% to prove a statistically significant change of IL-8 during EEN, and a power of 66% to detect differences in IL-10 between children with UC and non-IBD controls. Furthermore, we were unable to explore if the changes observed in peripheral blood were reflected in the mucosa. However, to our knowledge, this is the only study to consider EEN compliance, the impact of concurrent immunomodulatory treatments and, as discussed previously, to collect samples as participants finish EEN thus limiting the impact of food-reintroduction. Furthermore, our population was predominantly treatment-naïve with an immune profile less impacted by previous immune-modulation, a confounder that is more common in studies of an adult population.

In conclusion, distinct IRP profiles were observed both between patients with IBD and non-IBD controls and between patients with CD and UC, highlighting the role of Th1 cells and IFN-γ pathways in the pathogenesis of CD and Th17 cells in the pathogenesis of UC. Eight-week treatment with EEN was shown to modulate IRP profile, with these changes being more pronounced based on both disease location and if the patient achieved a significant FC response and by extension improved mucosal healing. Inflammation-related protein profile was able to differentiate IBD subtype and predict EEN treatment success, highlighting the potential of IRPs as diagnostic, treatment decision criterion and as biomarkers for monitoring disease activity.

## Supplementary Data

Supplementary data is available at *Inflammatory Bowel Diseases* online.

izae107_suppl_Supplementary_Materials
