# Supplementary material for: Inflammation-related Proteins Support Diagnosis of Inflammatory Bowel Disease and Are Modified by Exclusive Enteral Nutrition in Children With Crohn’s Disease, Especially of Ileal Phenotype
Source: Inflamm Bowel Dis. 2024 Jun 26;31(3):733–45. doi: 10.1093/ibd/izae107 (PMC11879199; doi:10.1093/ibd/izae107)
Supplement: izae107_suppl_Supplementary_Materials [file izae107_suppl_supplementary_materials.zip › izae107_Supplementary_Table_1.docx]

**Supplementary Table 1:** Evidence table of previous studies employing proximity extension assays to explore either differences in IRP profile between patients with IBD and non-IBD controls or the impact of induction treatments in active CD on IRP profile.

| **Study** | **Population** | **Findings which overlap with current study** | **Findings which do not overlap with current study** | **Percentage overlap** |
| --- | --- | --- | --- | --- |
| **Studies exploring differences in IRPs of patients with IBD compared to non-IBD controls** | | | | |
| Erik Andersson, 2017 (1) | Discovery cohort:  Adults with CD (n = 54, 69% in remission) Adults with UC (n = 54, 78% in remission) Adult non-IBD controls (n = 54)  Replication cohort: Adults with CD (n = 30, 80% in remission) Adults with UC (n = 30, 73% in remission) | IRPs different between CD and non-IBD: CXCL11, IL-18, IFN-γ, SCF, CCL-11, IL-17A, IL-6, CCL3 (MIP-1α), MMP-10, TNFSF14.  IRPs different between UC and non-IBD: OSM, CSF, CXCL9, CCL3, MMP-10.   IRPs different between CD and UC: FGF-19 | IRPs different between CD and non-IBD: IL-10RA, FGF-19, FGF-23, CASP-8, ENRAGE (S100-A12), TGF-α.  IRPs different between UC and non-IBD: TRANCE, CCL20, IL-5, CASP-8, ENRAGE (S100-A12), TGF-α, TNSF14. | IRPs different between CD and non-IBD: 59%  IRPs different between UC and non-IBD: 42%  IRPs different between CD and UC: 100% |
| Henrika Jodeleit, 2020 (2) | Adults with UC (n = 49, 61% in remission) Adult non-IBD controls (n = 23) | IRPs different between UC and non-IBD controls: MCP-3, CXCL9, HGF, CCL3 (MIP-1α), IL-8, CCL11, IL-17A, CCL19, IL-18R1, IL-6, OSM, CSF-1, VEGFA, FGF-21, MMP-10, DNER, IL-12B, OPG. | IRPs different between UC and non-IBD controls: CDCP1, CXCL10, CCL23, CXCL11, TGF-α, IL-10RB, IL-10, SLAMF1, IL-7, CD5, MCP-1, IL-18, MCP-4. | IRPs different between UC and non-IBD: 58% |
| Daniel Bergemalm, 2021 (3) | Discovery cohort:  Pre-diagnosis samples from adults with UC (n = 72)  Adult matched non-IBD controls (n = 140)   Inception cohort:  Adults with treatment naïve UC (n = 101)  Adult non-IBD controls (n = 50) | IRPs different between UC and non-IBD: CXCL9, CCL11, MMP-10. | IRPs different between UC and non-IBD: MCP-1, SLAMF1, CXCL11. | IRPs different between UC and non-IBD: 50% |
| Rahul Kalla, 2021 (4) | Adults with newly diagnosed active IBD (n = 328) Adult non-IBD controls (n = 224) | IRPs different between CD and non-IBD controls: CXCL9, OSM, HGF, CXCL11, IL-6, IL-8, IFN-α, IL-17A, CSF-1, VEGFA, SCF, MMP-1, DNER, CCL11, CCL23, MCP-3,   IRPs different between UC and non-IBD controls: IL-17A, IL-8, MMP-10, CXCL1, OSM, IL-17C, CXCL9, HGF, IL-6, CCL11, SCF, DNER  IRPs different between CD and UC: MMP-10, IL-17A. | IRPs different between CD and non-IBD controls: CCL20, IL-17C, CXCL1, IL-7, CXCL5, TRANCE, MCP-4, FGF-19, NT-3, TGF-α, TNFB  IRPs different between UC and non-IBD controls: CXCL11, TRANCE, CCL20, IL-10, TGF-α, | IRPs different between CD and non-IBD controls: 41% ^†^  IRPs different between UC and non-IBD controls: 71% ^†^  IRPs different between CD and UC: 100% ^†^ |
| Jie Chen, 2023 (5) | Discovery cohort: Adults with UC (n = 153)  Adult non-IBD controls (n = 293)   Validation cohort:  Adults with UC (n = 72)  Adult non-IBD controls (n = 140) | IRPs different between UC and non-IBD (discovery cohort): OSM, HGF, MMP-10, IL-6, CXCL1, CXCL9, CCL4.  IRPs different between UC and non-IBD (validation cohort): MMP-10, CXCL9, CCL11.   IRPs different between UC and non-IBD (pooled cohort): MMP-10, OSM, CXCL1, CXCL9, HGF, IL-6, CCL4, CCL11, IL-8. | IRPs different between UC and non-IBD (discovery cohort): TGF-α, TNFSF14, CCL20, ENRAGE.  IRPs different between UC and non-IBD (validation cohort): CXCL11, MCP-1.   IRPs different between UC and non-IBD (pooled cohort): TGF-α, TNFSF14, ENRAGE, CCL20, CASP8. | IRPs different between UC and non-IBD (discovery cohort): 64%  IRPs different between UC and non-IBD (validation cohort): 60%  IRPs different between UC and non-IBD (pooled cohort): 64% |
| Haim Leibovitzh, 2023 (6) | Pre-diagnosis samples from children with CD which are FDR of patients with CD (n = 71)   Healthy children which are FDR of patients with CD (n = 284) | IRPs associated with CD: CXCL9, OSM, CDCP1, CSF-1, HGF, MMP-10, CXCL11  IRPs associated with CD and FC levels: HGF, OSM, CSF-1, MMP-1 | IRPs associated with CD and FC levels: CXCL9, CDCP1, CXCL11, MMP-10, | IRPs associated with CD: 100% ^†^  IRPs associated with CD and FC levels: 50% ^†^ |
| **Studies exploring IRPs changes following treatment of active CD** | | | | |
| Stephanie Zwicker, 2017 (7) | Adults with IBD starting VDZ previously non-responsive to anti-TNF (n = 11) | IRPs which correlate with CRP: CCL4. | IRPs different in non-responders to VDZ and FC non-responders to EEN: MCP-4, IL-8  IRPs which correlate with FC: MCP-2  IRPs which correlate with CRP: CCL3, CCL23, CXCL1, CXCL5, CXCL9 | IRPs different in non-responders to VDZ and FC non-responders to EEN: 0%  IRPs which correlate with FC: 0%  IRPs which correlate with CRP: 17% |
| Maria M E Jongsma, 2023 (8) | Pediatric patients with active treatment naïve CD treated with either IFX (n = 48) or conventional treatment (EEN or oral prednisolone, n = 43). | IRPs different after treatment with IFX also found by us to be different after EEN: CCL23, CXCL10, TGF-α, FGF-21, MMP-1, Flt3L, IL-6, IL-24.   IRPs different after treatment with oral prednisolone also found by us to be different after EEN: IL-6, SCF, MMP-1, IL-24.   IRPs different after treatment with EEN: IL-24 | IRPs different after treatment with IFX also found by us to be different after EEN: CCL4, MCP-3, IL-17C, TNFSF14, ENRAGE, IL-8, CXCL9, IL-18, SIRT2, AXIN1, CASP8, ST1A1, HGF, CCL20, CCL3, IFN-γ, OSM, VEGFA, CDCP1, MMP-10, CXCL11.   IRPs different after treatment with oral prednisolone also found by us to be different after EEN: IL-17A, CD6, SLAMF1, IL12B, MMP-10, CD5, CCL20.  IRPs different after treatment with EEN: VEGFA, CDCP1, CXCL9, HGF, IFN-γ. | IRPs different after treatment with IFX also found by us to be different after EEN: 26%  IRPs different after treatment with oral prednisolone also found by us to be different after EEN: 36%  IRPs different after treatment with EEN: 17% |

Percentage overlap: The percentage of IRPs observed which were also observed within the current study. ^†^ Percentage excludes proteins which were included in a previous study but not included in the 92 IRPs we studied in the present cohort.

CD; Crohn’s disease, UC; Ulcerative colitis, IBD; Inflammatory bowel disease, IRP; Inflammation related protein, FDR; First degree relative, FC; Fecal calprotectin, IFX; Infliximab, VDZ; Vedolizumab.

References:

1. Andersson E, Bergemalm D, Kruse R, et al. Subphenotypes of inflammatory bowel disease are characterized by specific serum protein profiles. PLoS One. 2017; 12(10).

2. Jodeleit H, Milchram L, Soldo R, et al. Autoantibodies as diagnostic markers and potential drivers of inflammation in ulcerative colitis. PLoS One. 2020; 15(2).

3. Bergemalm D, Andersson E, Hultdin J, et al. Systemic Inflammation in Preclinical Ulcerative Colitis. Gastroenterology. 2021; 161(5):1526-39.

4. Kalla R, Adams AT, Bergemalm D, et al. Serum proteomic profiling at diagnosis predicts clinical course, and need for intensification of treatment in inflammatory bowel disease. J Crohn's Colitis. 2021;15(5):699-708.

5. Chen J, Zhou Y, Sun Y, et al. Bidirectional Mendelian Randomisation Analysis Provides Evidence for the Causal Involvement of Dysregulation of CXCL9, CCL11 and CASP8 in the Pathogenesis of Ulcerative Colitis. J Crohns Colitis. 2023;17(5):777-85.

6. Leibovitzh H, Lee SH, Raygoza Garay JA, et al. Immune response and barrier dysfunction-related proteomic signatures in preclinical phase of Crohn's disease highlight earliest events of pathogenesis. Gut. 2023;72(8):1462-71.

7. Zwicker S, Lira-Junior R, Höög C, et al. Systemic Chemokine Levels with "Gut-Specific" Vedolizumab in Patients with Inflammatory Bowel Disease-A Pilot Study. Int J Mol Sci. 2017;18(8).

8. Jongsma MME, Costes LMM, Tindemans I, et al. Serum Immune Profiling in Paediatric Crohn's Disease Demonstrates Stronger Immune Modulation With First-Line Infliximab Than Conventional Therapy and Pre-Treatment Profiles Predict Clinical Response to Both Treatments. J Crohn's Colitis. 2023;17(8):1262-77.
